# Dynamical sensitivity control of a single-spin quantum sensor

**DOI:** 10.1038/s41598-017-05387-w

**Published:** 2017-07-26

**Authors:** Andrii Lazariev, Silvia Arroyo-Camejo, Ganesh Rahane, Vinaya Kumar Kavatamane, Gopalakrishnan Balasubramanian

**Affiliations:** 1 MPRG Nanoscale Spin Imaging, Max Planck Institute for Biophysical Chemistry, Am Fassberg 11, 37077 Göttingen, Germany; 2 Department of NanoBiophotonics, Max Planck Institute for Biophysical Chemistry, Am Fassberg 11, 37077 Göttingen, Germany

## Abstract

The Nitrogen-Vacancy (NV) defect in diamond is a unique quantum system that offers precision sensing of nanoscale physical quantities at room temperature beyond the current state-of-the-art. The benchmark parameters for nanoscale magnetometry applications are sensitivity, spectral resolution, and dynamic range. Under realistic conditions the NV sensors controlled by conventional sensing schemes suffer from limitations of these parameters. Here we experimentally show a new method called dynamical sensitivity control (DYSCO) that boost the benchmark parameters and thus extends the practical applicability of the NV spin for nanoscale sensing. In contrast to conventional dynamical decoupling schemes, where *π* pulse trains toggle the spin precession abruptly, the DYSCO method allows for a smooth, analog modulation of the quantum probe’s sensitivity. Our method decouples frequency selectivity and spectral resolution unconstrained over the bandwidth (1.85 MHz–392 Hz in our experiments). Using DYSCO we demonstrate high-accuracy NV magnetometry without |2*π*| ambiguities, an enhancement of the dynamic range by a factor of 4 · 10^3^, and interrogation times exceeding 2 ms in off-the-shelf diamond. In a broader perspective the DYSCO method provides a handle on the inherent dynamics of quantum systems offering decisive advantages for NV centre based applications notably in quantum information and single molecule NMR/MRI.

## Introduction

A solid-state quantum system operable under varied conditions finds immense use in precision sensing. The electron spin associated with nitrogen-vacancy defects in diamond has robust coherence properties and permits quantum metrology at the nanoscale^1–4^. Despite cutting edge advances using NV sensors for probing nanoscale physical quantities, a few factors still repress their widespread adaptations^5–8^. Some of those impediments are the required foreknowledge/control of the quantity to be measured, ruinous effects of environmental noise, limited dynamic range (DR), and ambiguous frequency information.

Conventional NV precision metrology schemes are interferometric methods based on measuring the phase evolution of the spin’s superposition state during a defined free precession interval (*τ*)^1, 3, 9–11^. The nature of such conventional phase acquisition techniques results in a |2*π*| ambiguity of the signal, thus for precision measurements a control of the external field or prior information on the magnitude is necessary^12, 13^. Dynamical Decoupling (DD) schemes prolong the net phase accumulation to the spin-spin relaxation time *T*_2_ by applying series of timed *N* · *π* pulses interleaved within free-precession intervals^10, 14–16^. Noise spectroscopy and nuclear spin sensing based on DD schemes are usually performed by varying the free-precession time *τ* and thereby profiling the noise spectral density corresponding to *f*_*s*_ = 1/[4 · (*t*_*π*_ + *τ*)] with frequency resolution given by Δ*f*_*s*_ = 1/[*N* · 4 · (2*τ* + *t*_*π*_)]^17, 18^. This has been employed to sense nuclear spin noise employing NV spins^19–21^. In this time-domain sensing scheme the sensing frequency and spectral resolution are strictly correlated: higher(lower) frequencies are sensed with low(high) spectral resolution. In situations dominated by high frequency and broad band noise the poor spectral selectivity results in loss of coherence. Increasing the DD order (*N* · *π*) results in ambiguous frequencies, further complicated by harmonics^17, 22, 23^. These shortcoming are often encountered with near surface NV spins^24, 25^ and other quantum systems that are housed amidst heterogeneous interactions^26–28^.

Here we demonstrate the method of dynamical sensitivity control (DYSCO) along with its application in NV metrology that mitigates a large part of the hurdles mentioned above. The DYSCO method in addition to boosting the DR of the sensor to 4 · 10^3^ also enables a temporal modulation of the sensor in a piecewise manner with desired sensitivities, and thus permits to retrieve interactions in the frequency domain. This is a unique property of our approach. We show this method allows nuclear spin sensing in the frequency-domain with wide-bandwidth without compromising the spectral resolution. Applications of spin-based molecular scale NMR/MRI in which NV centres reside close to surfaces yet require high sensitivity and frequency selectivity^20, 21^ would directly benefit from employing the DYSCO sensing scheme^29, 30^. The method is in principle applicable to any qubit system influenced by noisy surroundings^26–28^. It should be of use in numerous solid-state quantum architectures to identify spin resources^31, 32^, possible dissipation pathways^33, 34^ and routes to protecting the qubits^35, 36^. The piece-wise dynamical sensitivity control provides an alternative scheme to decouple the central spin from inherent noise and realize high-fidelity quantum operations in the solid-state^37, 38^.

## Results

**Figure 1:**
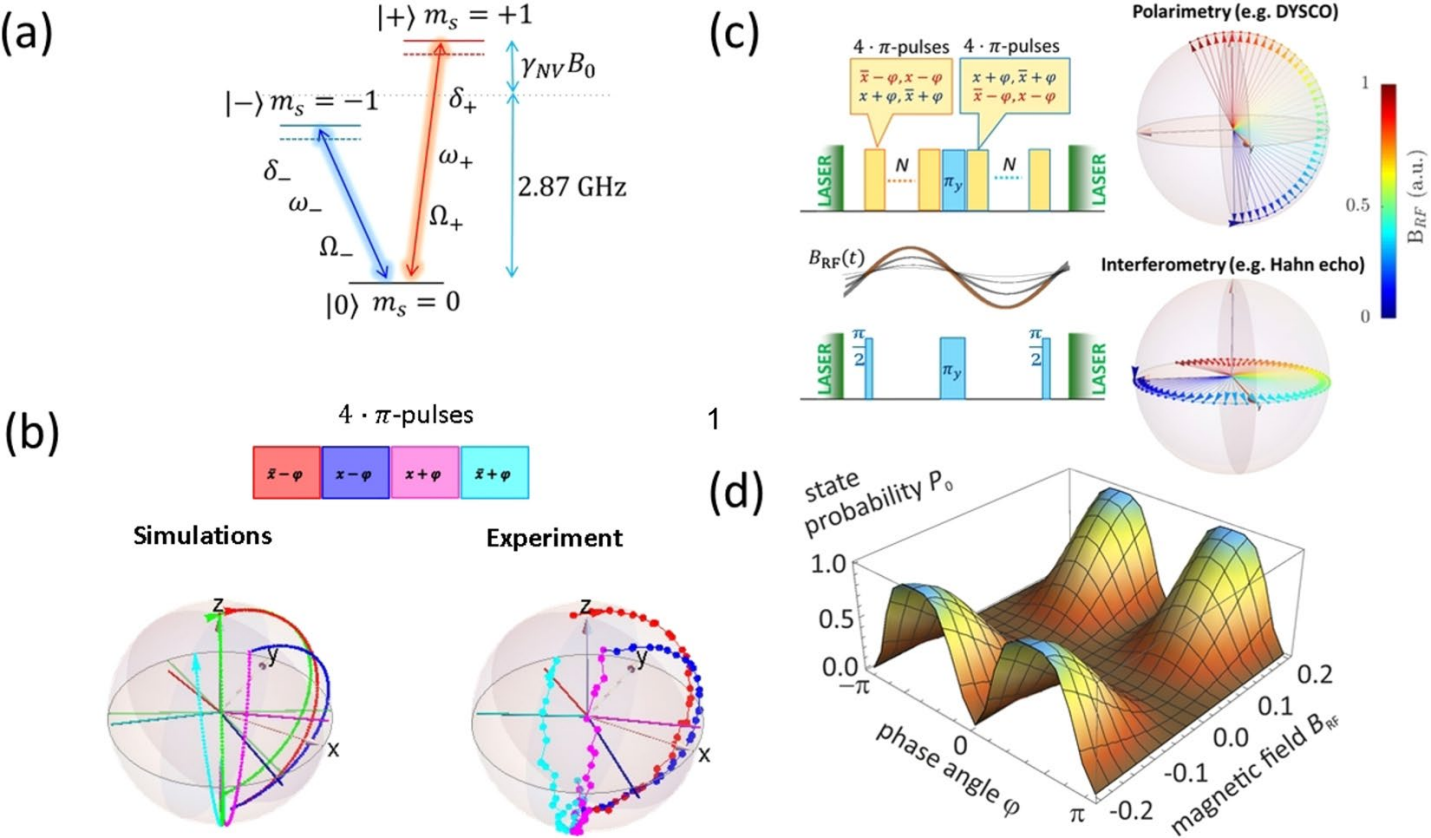
NV centre system and Dynamical sensitivity control (DYSCO) spin dynamics. (**a**) Energy level scheme of the NV centre an electron spin in the ground state triplet. (**b**) Top: Schematic representation of a DYSCO 4 · *π*-pulse unit. Bottom: Simulations and experimentally traced spin vector trajectory for one 4 · *π*-pulse unit represented on the Bloch sphere spanned by |0〉 and |−〉. The green trajectory in simulations depicts spin evolution when sequentially driven by 4 · *π*-pulse and vanishing RF field *B*_RF_ = 0. The coloured trajectories in the simulations and experiment are shown for *B*_RF_ ≠ 0. Individual *π*-pulses are shown in red, blue, magenta and cyan dots and traces. (**c**) Schematic of the DYSCO method and the expected changes in the final state population *P*_0_ for different values of the *B*_RF_ field amplitude shown in comparison to the Hahn-echo sensing method (note the last *π*/2 pulse used in NV-metrology based on free-precession/interferometry (bottom) is not required in the DYSCO scheme (top) distinguishing the sensing mechanism of DYSCO). (**d**) Explicit calculation of the level occupancy *P*_0_ and its dependence on the *B*_RF_ field and the phase angle *φ* of the driving pulses at the end of the pulse sequence for *N* = *1* (the *B*_RF_ field is given in units of Ω_−_).

Specifically, we wish to measure a weak RF magnetic field *B*_RF_(*t*). This is of immediate practical relevance in nuclear magnetic resonance (NMR) and magnetic resonance imaging (MRI) applications, where the *B*_RF_(*t*) field originates from precessing nuclear spins. As spin probe, we employ the NV centre ground state triplet system featuring the spin states |0〉, |−〉 and |+〉 (details in Supplementary Information S1). For these sensing experiments we address single, isolated NV defects housed in a high-purity CVD diamond (Element Six). Details about the NV-spin manipulation and the employed experimental setup are provided in the Methods section. The NV spin is coherently driven on the |0〉 to |−〉 transition by a microwave field (MW) of amplitude *B*_1_, detuning *δ*_−_ and phase *φ* (cf. Fig. 1(a)). In the presence of an external weak RF magnetic field *B*_RF_(*t*) the system interaction Hamiltonian (after the rotating wave approximation) has the form

1 $$H(t)=-{\delta }_{-}|-\rangle \langle -|-{\gamma }_{{\rm{N}}{\rm{V}}}{B}_{{\rm{R}}{\rm{F}}}(t)\cdot (|+\rangle \langle +|-|-\rangle \langle -|)-\frac{{{\rm{\Omega }}}_{-}}{2}({e}^{i\phi (t)}|0\rangle \langle -|+{\rm{h}}.{\rm{c}}.),$$

where *γ*_NV_ ≈ −2*π* · 28 GHz/T is the NV gyromagnetic ratio and Ω_−_ = −*γ*_NV_ *B*_1_ is the Rabi frequency of the driving microwave field with the phase *φ*.

### The DYSCO pulse sequence

The dynamical sensitivity control scheme is composed of *N* control units each consisting of 4 · *π*-pulses that sequentially drive the NV spin state in a phase alternating manner. Figure 1(b) shows an illustration of the basic 4 · *π*-pulse unit used to compose the DYSCO sequence:

2 $${[{\pi }_{\bar{x}-\phi },{\pi }_{x-\phi },{\pi }_{x+\phi },{\pi }_{\bar{x}+\phi }]}^{N}-{\pi }_{y}-{[{\pi }_{x+\phi },{\pi }_{\bar{x}+\phi },{\pi }_{\bar{x}-\phi },{\pi }_{x-\phi }]}^{N},$$

where $\bar{x}$ denotes the axis anti-parallel to the *x* axis. The total evolution time is given by *t*_*N*_ = (4_*N*_ + 1/2) · 2*π*/Ω_−_ and its reciprocal defines the spectral resolution. In one functional 4 · *π*-pulse DYSCO unit, each pair of pulses that are applied at diametrically opposite axis ${\pi }_{\bar{x}-\phi },{\pi }_{x-\phi }\,{\rm{and}}\,{\pi }_{x+\phi },{\pi }_{\bar{x}+\phi }$ accumulates the influence of *B*_RF_ while the phase shift *φ* between these pulse pairs controls the net influence that is encoded into the population. A *π*_*y*_-pulse placed in the middle of the sequence followed by *N* reversely ordered 4 · *π*-pulses is designed to compensate for pulse-errors.

Unlike conventional sensing schemes the DYSCO pulse scheme is not based on a magnetic field dependent phase acquisition in a superposition state, but instead it directly encodes a magnetic field dependent shift on the spin state population (cf. Fig. 1(c)). The magnitude of the population change can be varied by a control parameter of the microwave field that drives the spin. This provides a means to modulate the sensitivity of a single spin to the external field circumventing free-precession. In optical terminology, this method is analogous to a polarimetric measurement scheme based on optical rotations that complements the interferometric methods employed in conventional, free-precession based sensing (cf. Fig. 1(c)). In the context of metrology, polarimetry schemes are generally considered to be robust against drifts and fluctuations^39^.

### Dynamically controlling the sensitivity of a single spin

**Figure 2:**
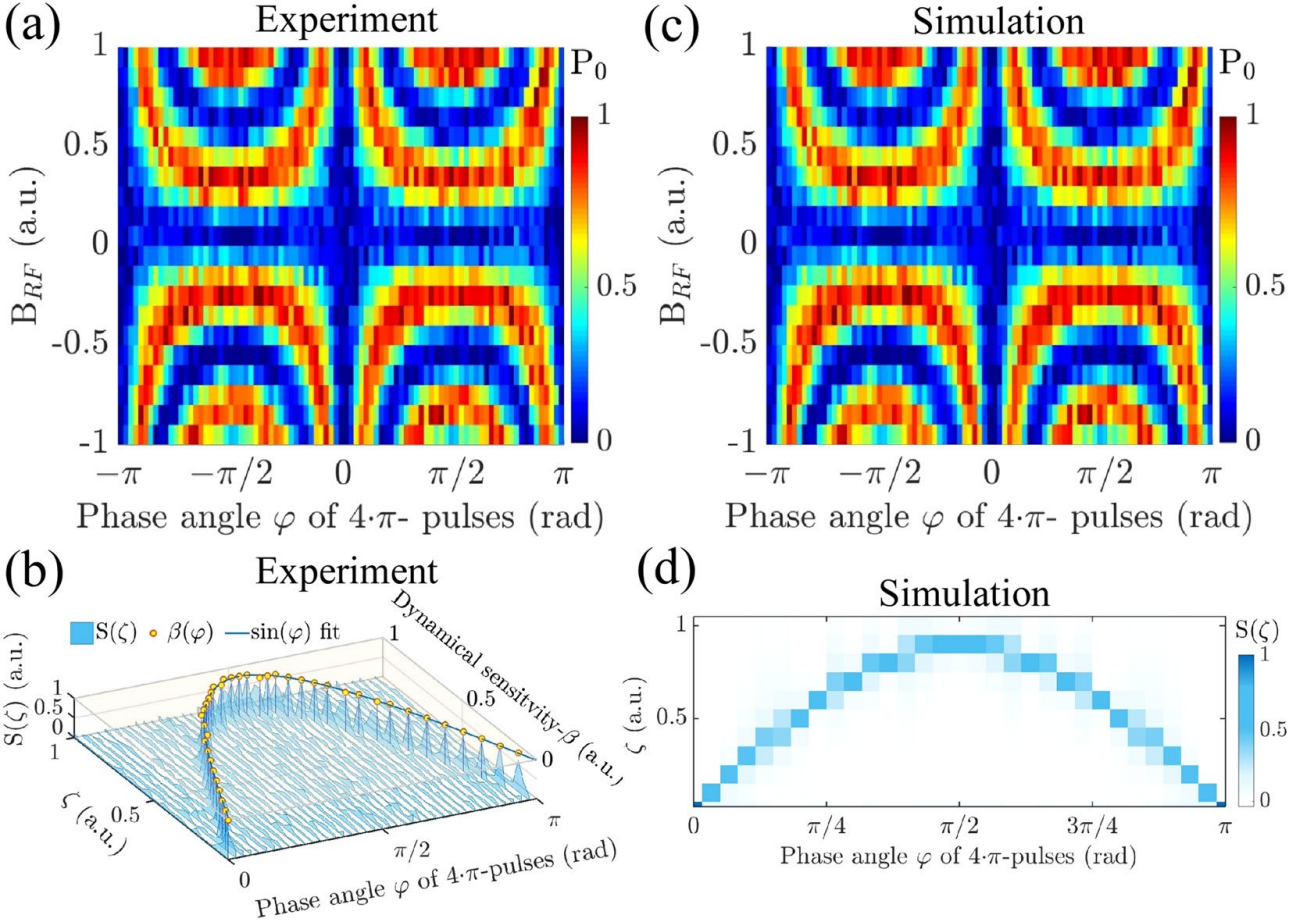
Results of dynamical sensitivity control. (**a**) Experimental results showing the dependence of state population *P*_0_ as a function of *B*_RF_ and *φ* for *N* = 20. (**b**) Simulations showing the *P*_0_ as a function of *B*_RF_ and *φ* for *N* = 20 matching the experiments shown in (**a**). (**c**) Experimental results showing *S*(*ζ*), the dynamical sensitivity *β*(*φ*), and its dependence on the 4 · *π*-pulse unit phase angle *φ* for *N* = 160. (**d**) The simulation results of *S*(*ζ*) dependence on *φ* obtained matching the experimental conditions shown in (**c**).

To demonstrate that the sensitivity of a quantum sensor can be controlled, we drive the NV-spin using the DYSCO pulse sequence and simultaneously subject the spin to an external field *B*_RF_. Employing a relatively short DYSCO sequence with *N* = 20 of the 4 · *π*-pulse units we study the dependence of *P*_0_(*φ*, *B*_RF_) for *φ* ∈ [−*π*, *π*]. We find that the population *P*_0_(*φ*, *B*_RF_) responds harmonically in the range $0 < \gamma {B}_{{\rm{RF}}}\le \frac{{{\rm{\Omega }}}_{-}}{4}$ while *φ* controls the rate of oscillations. The explicit theoretical calculations shown in Fig. 1(d) (for details see Supplementary Information S2) agree with the experimental results shown in Fig. 2(a) and the numerical simulations displayed in Fig. 2(b).

The sensitivity of a measurement is given by the smallest change in the quantity to be measured (here *δB*_RF_) that still leads to a resolvable change of the experimental observable (here *δP*_0_)^1, 9^. Both in the case of conventional and DYSCO magnetometry the population *P*_0_(*B*_RF_) varies harmonically with increasing magnetic field amplitude *B*_RF_, and the sensitivity can be obtained from the maximum slope of the response d*P*_0_/d*B*_RF_. As the signal is harmonic, the Fourier transform $( {\mathcal F} )$ of the population with respect to *B*_RF_ given by $S(\zeta )\propto  {\mathcal F} ({P}_{0}({B}_{{\rm{RF}}}))$ features a single Dirac delta distributed *ζ*-component which is a measure for the sensitivity. We obtain the relation between the phase control and the sensitivity of the sensor employing *N* = 160 of the 4 · *π*-pulse units and measuring the state occupancy *P*_0_(*φ*, *B*_RF_) for *φ* ∈ [0, *π*]. As the signal *P*_0_(*φ*, *B*_RF_) is harmonic in the *B*_RF_ range, we show its corresponding Fourier transform *S*(*ζ*) for every value of *φ* ∈ [0, *π*] in Fig. 2(c) (experiment). Thus, the sensitivity dependence of the DYSCO sequence can be deduced from the experimental data as

3 $$\beta (\phi )\propto |\mathop{{\rm{\arg }}\,{\rm{\max }}}\limits_{\zeta }[{ {\mathcal F} ({P}_{0}({B}_{{\rm{RF}}}))|}_{\phi }]|.$$

In the following, we denote *β*(*φ*) as the ‘dynamical sensitivity’, given that this quantity can be continuously varied in analog manner as desired through the phase angle *φ* of the 4 · *π* pulse units. Figure 2(c) and (d) illustrates the significance of the function *β*(*φ*) in the context of magnetometery. The value *β* = 0 denotes a phase condition *φ* = 0 that sets a DYSCO unit insensitive (or minimally sensitive) to external *B*_RF_ field while *β* = 1 defines a condition that makes a DYSCO unit most sensitive (high sensitivity) to the *B*_RF_ field. Empirically we find that the dynamical sensitivity has a dependence on the MW phase described by *β*(*φ*) ∝ |sin *φ*|.

### Numerical simulations and analytical results

For the experimentally relevant case involving many pulse units (*N* > 100) we used the Mathematica module SpinDynamica^40^ to compute the response of the NV spin on the *B*_RF_ field and the phase angle of the pulse sequence. The simulation results of the spin state dependence of *P*_0_(*φ*, *B*_RF_) for *N* = 20 of the 4 · *π*-pulse units in the range *φ* ∈ [−*π*, *π*] are presented in Fig. 2(b) and match the experimental results shown in Fig. 2(a) very well. Similarly the computed dependence of *S*(*ζ*) for *φ* ∈ [0, *π*] using *N* = 160 of the 4 · *π*-pulse units shown in Fig. 2(d) matches with the experimental outcome displayed in Fig. 2(c) (for details see Supplementary Information S2).

In the two-dimensional subspace spanned by |0〉 and |−〉 the spin vector dynamics can be represented by rotation matrices. Using successive rotations corresponding to the DYSCO pulse sequence we derive an explicit expression for *P*_0_(*φ*, *B*_RF_). From the series expansion of *P*_0_(*φ*, *B*_RF_) we obtain an analytical expression for the dynamical sensitivity as $\beta (\phi )\propto (\frac{{B}_{{\rm{RF}}}}{{B}_{1}}\,\sin \,\phi )$. This is consistent with the experimental results (details in Supplementary Information S3). We observe that the resultant population shifting effect of the DYSCO sequence is almost identical when the spin state is initially prepared in a superposition state (see Supplementary Information S2ii). For an intuitive understanding an illustrative representation of the spin dynamics in terms of rotations on the Bloch sphere is detailed in Supplementary Information S3i.

### Quantitative DYSCO magnetometry of arbitrary AC fields

We show the piecewise dynamical control of the sensitivity of a spin sensor allows us to measure strength and frequency components of arbitrary oscillatory fields (details in Methods). In this spectroscopy approach the desired spectral resolution specifies the required signal acquisition time *t*_*N*_. A number of *N* times 4 · *π*-pulse units are concatenated for the total duration *t*_*N*_. Each of these 4 · *π*-pulse units can be composed to modulate the instantaneous dynamical sensitivity of the NV spin sensor to match any arbitrary temporal profile of the signal *f*_RF_ to be sensed. This piece-wise sensitivity control using discretized units enables frequency resolved sensing (spectroscopy) of arbitrary oscillating magnetic fields.

**Figure 3:**
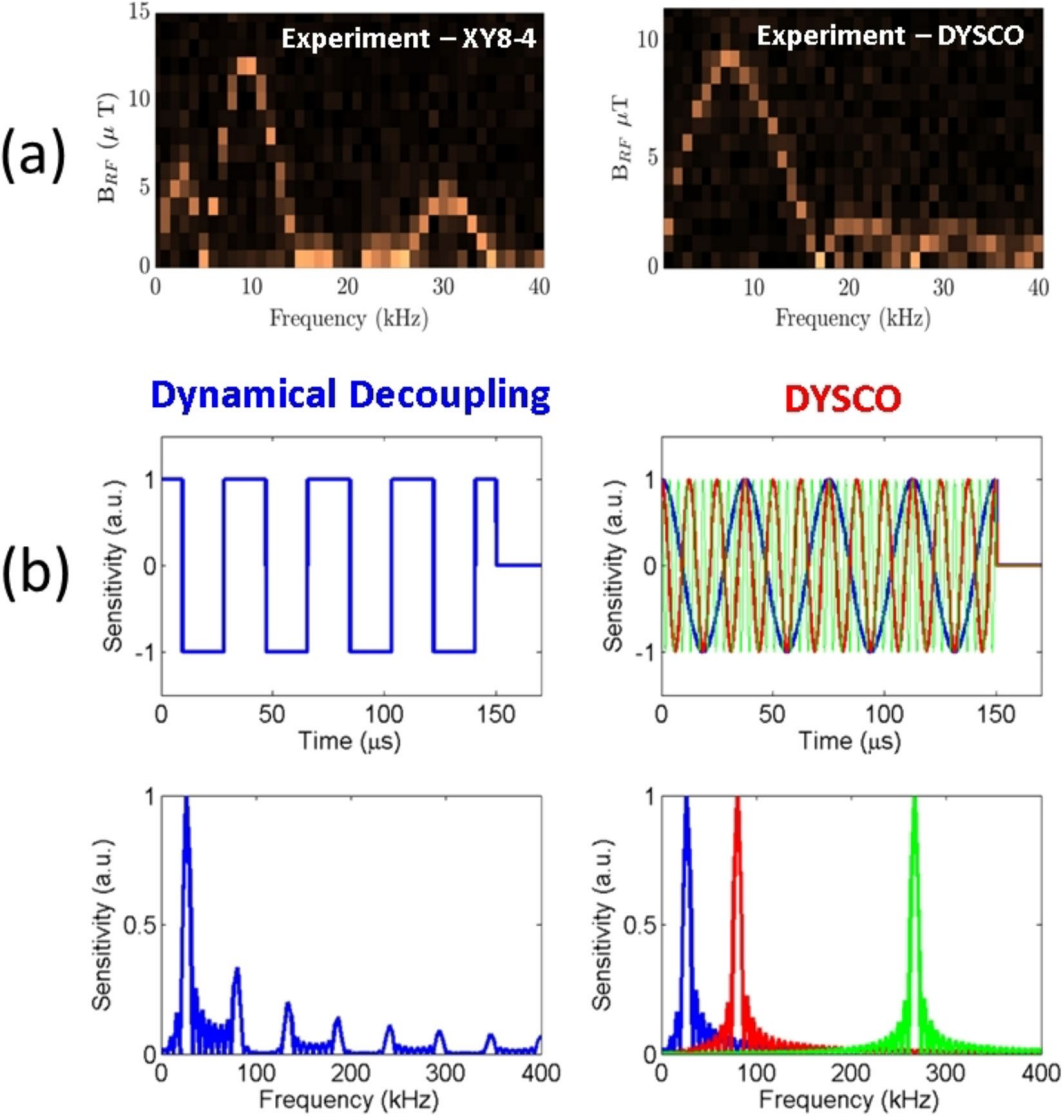
Frequency resolved magnetometry (spectroscopy) and contrast between the XY8-4 dynamical decoupling and the DYSCO scheme. (**a**) Experimental results of magnetic field sensing of a 8 kHz phase-synchronized RF signal using a conventional multi-pulse scheme (left) and using the DYSCO method (right).(**b**) Free precession based multi-pulse sequences (top left) produce harmonics in their filter function (bottom left), while the DYSCO sequence (top right) produces a single frequency response (bottom right). The frequency response corresponding to three different DYSCO modulation frequencies is shown in colour code.

The DYSCO magnetometry spectra are recorded measuring the NV spin state occupation *P*_0_ and varying both the modulation frequency of the dynamical sensitivity *β*(*f*_*s*_) and the maximum value of dynamical sensitivity *β*_*k*_ separately. The sensor shows a distinct signature when the modulation frequency of the dynamic control *f*_*s*_ matches the frequency *f*_RF_ of the external *B*_RF_ field. At that modulation frequency *f*_*s*_ upon variation of the maximum sensitivity *β*_*k*_ the resulting ${\mathcal F} ({P}_{0}({\beta }_{k}))$ response gives a measure of the magnetic field strength (cf. Fig. 3(a)). If the signals are phase synchronized, the spin state oscillates with a rate proportional to the strength of the magnetic field *B*_RF_. If the external field happened to be asynchronous and when *β*_*k*_ is varied the signal gradually drops from *P*_0_ = 1 and reaches a value of half the visibility *P*_0_ = 1/2.

In Fig. 3(a), we present the experimental results of the NV sensor response to an external field oscillating at 8 kHz. The frequency resolved magnetometry obtained using conventional multi-pulse interferometric schemes (here, e.g., a XY8 sequence) exhibits harmonic responses (cf. Fig. 3(a) left) corresponding to its characteristic filter function(cf. Fig. 3(b) left). In marked contrast to this, the DYSCO method precludes harmonic responses in the filter function (cf. Fig. 3(b) right). When the same experiment is performed using DYSCO spectroscopy by an analog modulation of the dynamical sensitivity in the desired frequency band we obtain a response only at the applied RF field frequency (cf. Fig. 3(a) right).

**Figure 4:**
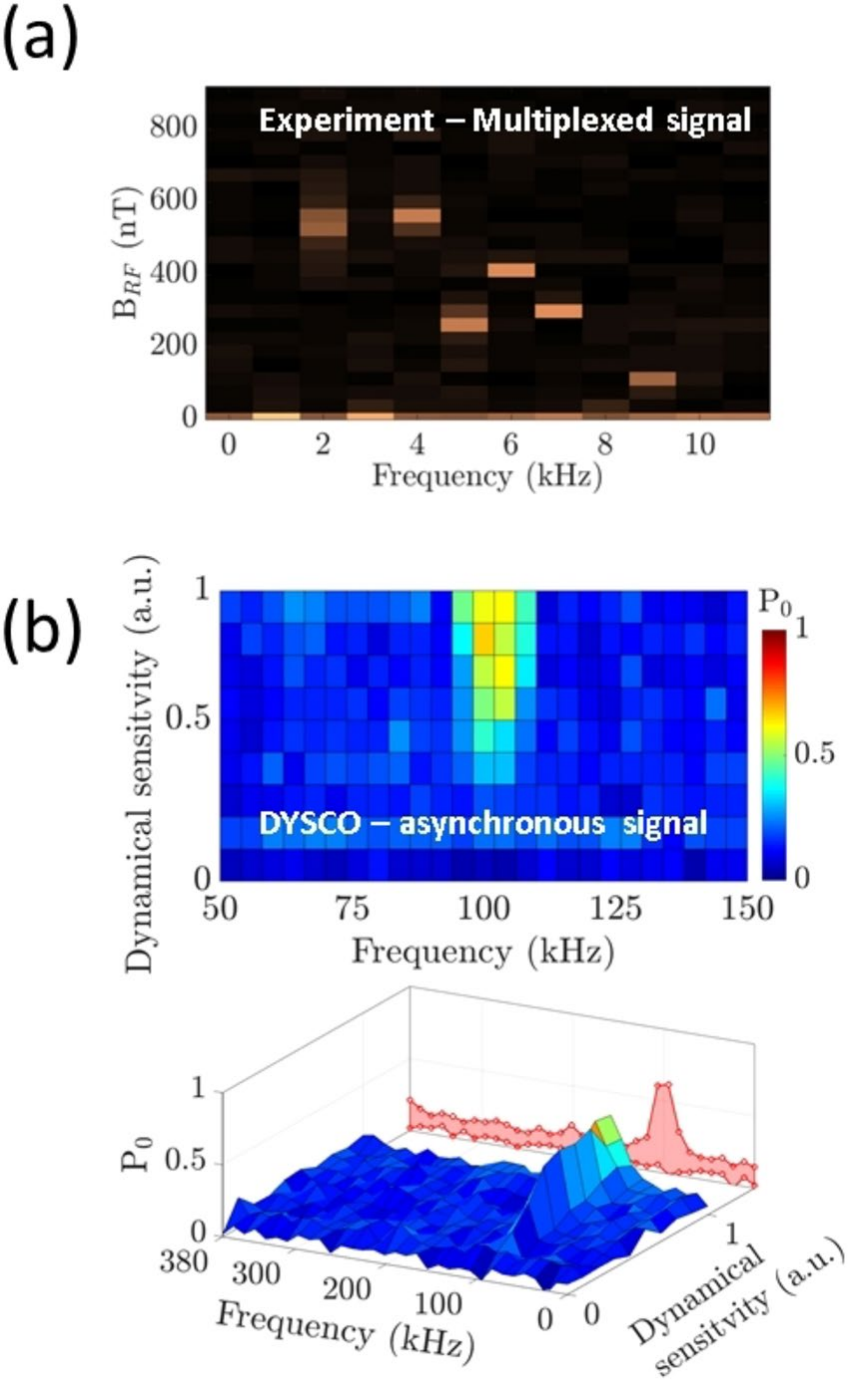
Spectroscopy using dynamical spin control. (**a**) Experimental DYSCO spectrum of an RF signal consisting of six frequency components sensed with 1 kHz spectral resolution. (**b**) Frequency domain response of an asynchronous 100 kHz noise signal.

The DYSCO method is useful for resolving frequency components of multiplexed signals, because the sensitivity can be tuned in a controlled manner to detect signals with narrow bandwidth. In the results shown in Fig. 4(a), we inject a set of six frequencies (2, 4, 5, 6, 7, and 9 kHz) of corresponding relative strengths (1.0, 1.0, 0.5, 0.75, 0.55 and 0.2) and measure the response in a spectrogram fashion with a resolution of 1 kHz.

Probing phase asynchronous signals is particularly interesting in the context of sensing nuclear spin noise. We realized this asynchronous measurement scheme as before by dynamically modulating the sensitivity *β*(*t*_*n*_) with a desired frequency *f*_*s*_ and gradually varying the maximum of the dynamical sensitivity *β*_*k*_ in steps (details in Methods). A phase asynchronous signal with a frequency of 100 kHz is injected into a micro-coil, and the spectral response of the NV spin *P*_0_ is plotted in Fig. 4(b) as colour coded spectrogram. At the maximum response, the spin state population attains a value of *P*_0_ = 1/2 indicating incoherent interaction. The results also exhibit the absence of harmonic responses (cf. Fig. 4(b)).

### Harmonics-free spin noise spectroscopy

**Figure 5:**
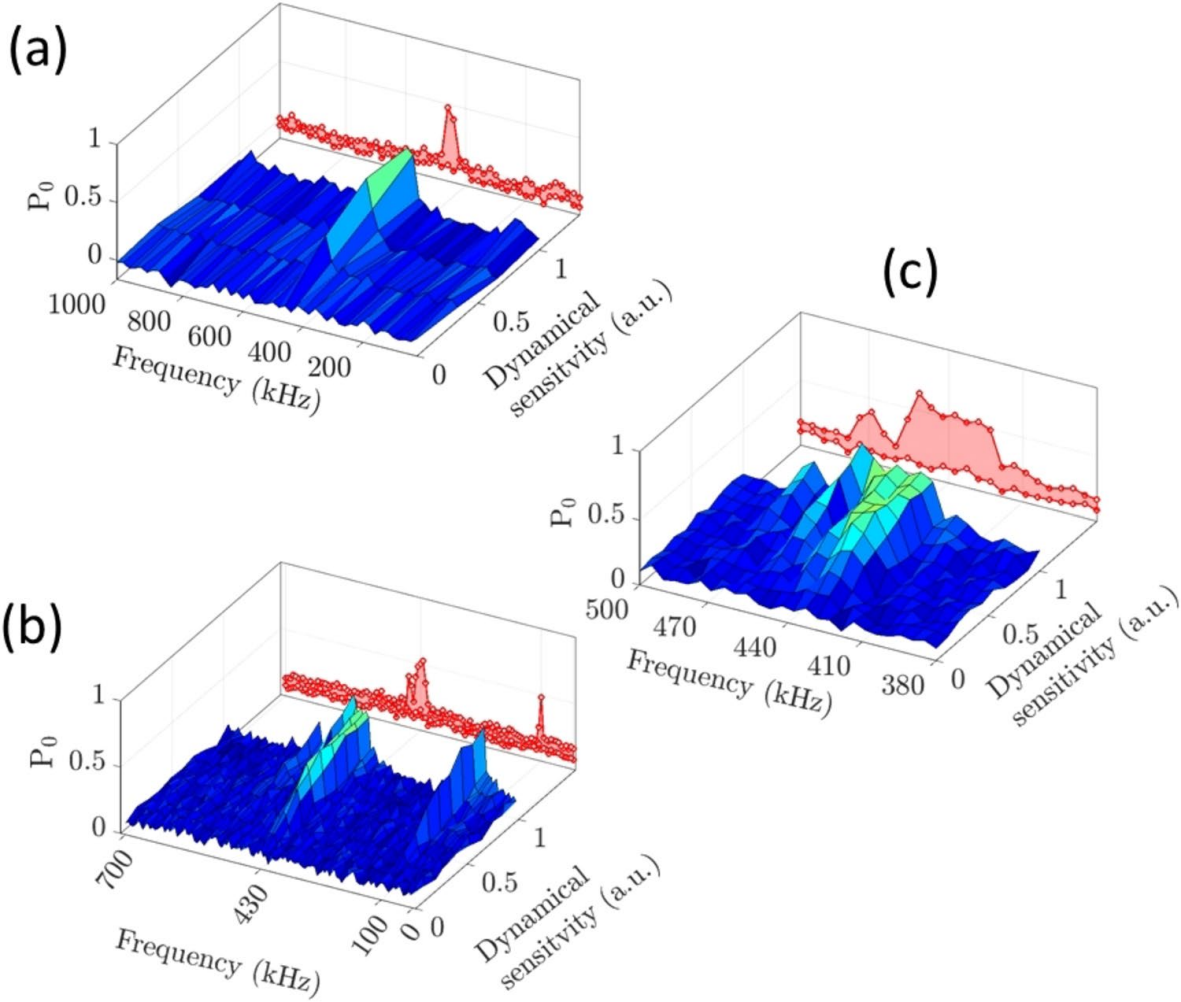
High-resolution DYSCO noise spectrum of ^13^C nuclear spin bath. (**a**) The spin noise spectrum of the ^13^C nuclear spin bath shows distinctly the carbon Larmor frequency in a wide range without any harmonics. (**b**) The ^13^C noise spectrum together with 100 kHz noise signal showing no undesired responses. (**c**) Finer resolved spectrum with a resolution of 5 kHz showing signatures of ^13^C spins being weakly coupled to the NV spin with a strength of 60 kHz.

In another set of experiments, we demonstrate the use of the DYSCO method to probe noise spectral details of the ^13^C spin bath in the vicinity of a single NV spin. The ^13^C nuclear spins in the bath precess with a certain spread in Larmor frequencies. This causes asynchronous magnetic field fluctuations to influence the NV spin. As detailed above we modulate the NV spin’s dynamical sensitivity in an extended range from 10 kHz to 1000 kHz with 10 kHz resolution and record the response (cf. Fig. 5(a)). A high-resolution scan in the frequency range from 380 kHz to 500 kHz near the ^13^C Larmor frequency shows well-resolved signatures of the NV spin coupled to distant ^13^C nuclear spins with a coupling strength as small as 60 kHz (Fig. 5(b)).

It is evident from Fig. 5(a) that the ^13^C nuclear spin noise signatures are seen only at the ^13^C Larmor frequency (430 kHz), without any harmonic or spurious artefacts. To emphasize the absence of harmonics, we injected an additional 100 kHz RF asynchronous signal and observed the response at the expected frequencies. Moreover, the injected signal at 100 kHz did not influence the ^13^C Larmor response occurring roughly at the 4th harmonic of the injected signal (Fig. 5(b)). The frequency resolved method using DYSCO thus permits sensing ^1^H nuclear spins without interference from ^13^C nuclear spins that precess at 1/4 the Larmor frequency of ^1^H spins $({\gamma }_{{\rm{1H}}}/{\gamma }_{13{\rm{C}}}\approx 4)$.

### Prolongation of interrogation time

**Figure 6:**
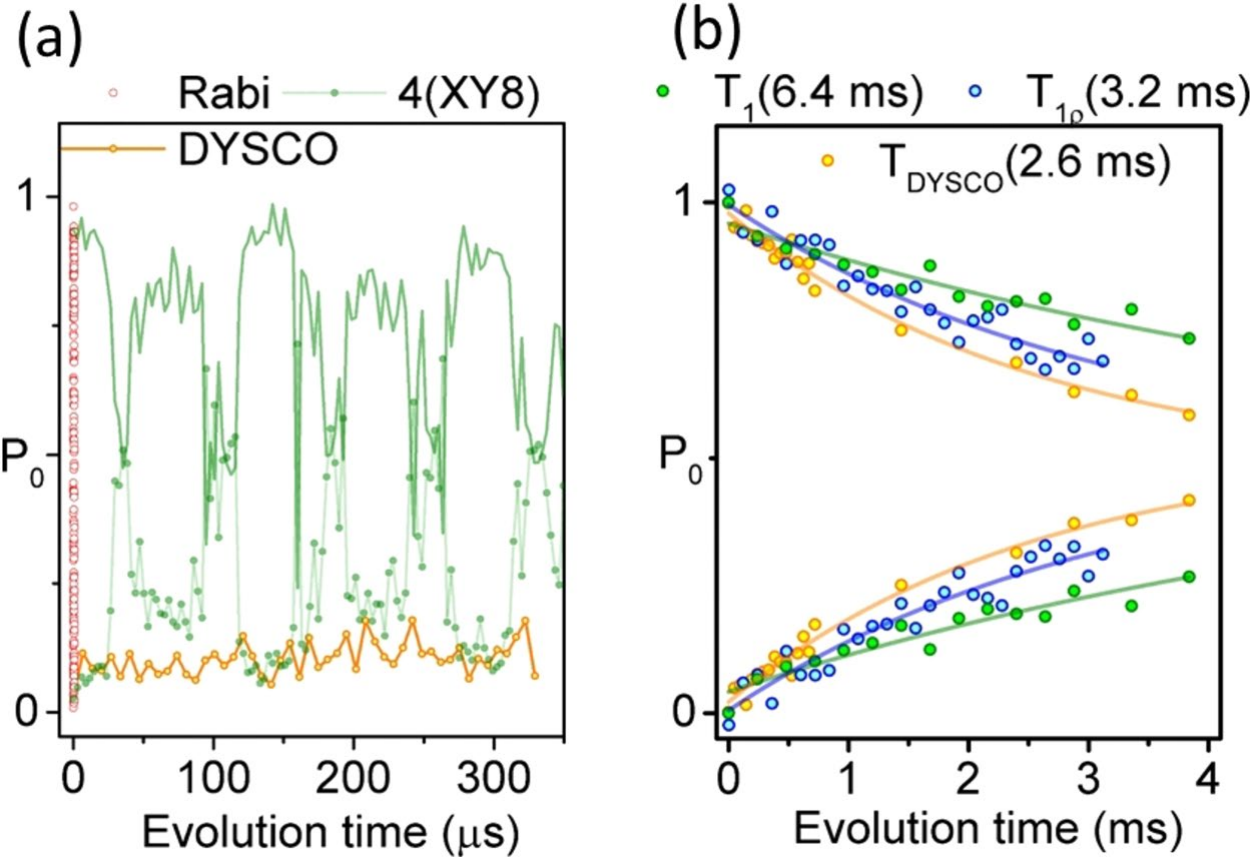
Interrogation time of DYSCO compared to XY8-4 dynamical decoupling. (**a**) Experimental results of an XY8-4 dynamical decoupling measurement together with a DYSCO measurement (without modulations) clearly showing the absence of characteristic ^13^C nuclear spin influences. (**b**) Experimental data and fits showing the relaxation times *T*_1_, *T*_1_ *ρ* and *T*_DYSCO_ of a single NV spin.

As an additional advantage for NV magnetometry, we show the frequency selectivity of the dynamical control allows sensing without being compromised by ^13^C spin-bath signatures (cf. Fig. 6(a)). The coherence of the NV system when driven by a DD sequence XY8-4 as a function of the interpulse delay (*τ*) is given in Fig. 6(a) (green). This shows collapses and revivals corresponding to the NV spin coupling to several ^13^C nuclear spins of various coupling strengths. This is primarily because the XY8-4 sequence senses the interactions in the time-domain, so several frequency components manifest their signature at any interrogation time (8 · 4 · *τ*). On the other hand, the behavior of the spin state (*m*_*s*_ = −1) manipulated by DYSCO (orange) is unperturbed by the carbon influences because the frequency to be sensed is set to be different from the Larmor frequency of ^13^C nuclear spins. Hence, the spin interrogation time is extended despite the NV defect being housed in a natural abundance diamond sample. For reference we plot a few Rabi oscillations (red open circles) as a measure of the contrast (${P}_{{m}_{s}=0}$ and ${P}_{{m}_{s}=-1}$) of the quantum system. Figure 6(b) indicates the spin state (*m*_*s*_ = −0 and *m*_*s*_ = −1) decay under the influence of the DYSCO pulse sequence (orange) on longer interrogation times and also includes experimental results of *T*_1_ (green) and *T*_1*ρ*_ (blue) of the NV spin.

These measurements show that by using standard electronic grade diamond (1.1% ^13^C) the DYSCO scheme provides an elegant route to achieve longer interrogation times to a maximum of *T*_DYSCO_ = 2.55 ms that approaches the spin relaxation time in the rotating frame *T*_1*ρ*_ = 3.2 ms (Fig. 6(b)). This significant improvement corresponds to an enhancement in the sensitivity that was previously considered exclusive to ^12^C isotope enriched diamond. The DYSCO method permits the NV sensor to operate over a wide frequency bandwidths ranging from $\frac{{{\rm{\Omega }}}_{-}}{9\pi }$ to 1/*T*_DYSCO_ limited by the minimum (4*N* + 1/2) · 2*π*/Ω_−_ and maximum permissible interrogation time *T*_DYSCO_ of the sensing scheme *t*_*N*_.

### Boosting the dynamic range of the NV sensor

**Figure 7:**
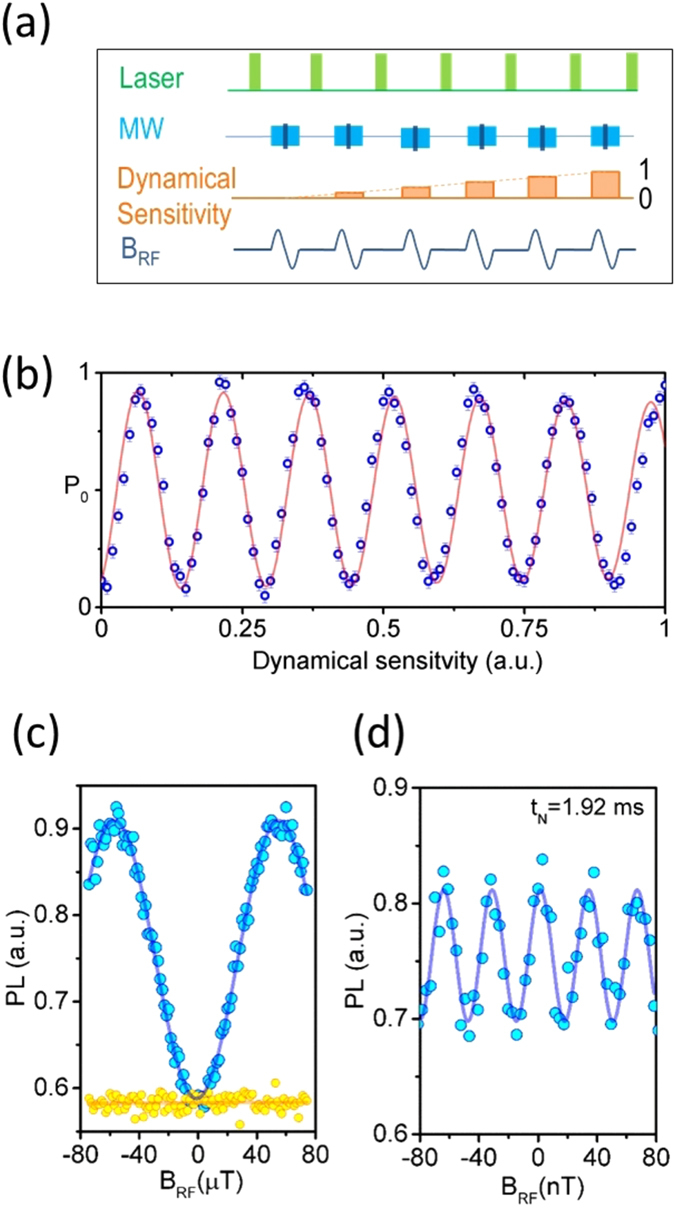
DYSCO magnetometry and dynamic range enhancements. (**a**) Schematic and (**b**) experimental results of magnetic sensing obtained without |2*π*| ambiguity by controlling the dynamical sensitivity of the NV spin. (**c**) Results depicting the extent of the dynamic range (4 · 10^3^) achieved using DYSCO. The lower bound is obtained by setting *β* = 1 (blue curve), whereas *β* = 0 (yellow curve) provides an upper bound for the DR. (**d**) Precision magnetometry performed at *t*_*N*_ = 1.92 ms total acquisition time using an NV spin present in a natural abundance 1.1% ^13^C diamond.

The dynamic range (DR) is the extent of the magnetic field that can be measured without |2*π*| ambiguities arising from the oscillatory nature of the *P*_0_(*B*_RF_) response. The DYSCO method presented here gives us the ability to gradually ramp the dynamical sensitivity of the spin *β*_*k*_ between 0 and 1 in arbitrary steps and to measure the magnetic field *B*_RF_ influence on the NV spin free from |2*π*| ambiguity (cf. Fig. 7(a)). The experimental results are shown in Fig. 7(b), where the spin-state as a function of the *β*_*k*_ oscillates at a rate proportional to the magnetic field. The permissible bounds of the DR are given by the ratio of the largest and smallest *B*_RF_ field changes that can be measured by the sensor. For a single spin magnetometer we achieve a DR of about 4 · 10^3^, obtained from the ratio of the respective maximum slopes of the *P*_0_(*B*_RF_)|_*φ*→0_ and the *P*_0_(*B*_RF_)|_*φ*→π/2_ responses shown in Fig. 7(c). The ratio of the *B*_RF_ scales in Fig. 7(c) and (d) could be also seen as an indicator for the achieved boost in dynamic range of the NV sensor. Parameters that bound the dynamical control of the NV spin are the Rabi frequency Ω_−_ = *γ*_*e*_ *B*_1_ and the maximal interrogation time *T*_DYSCO_. Under our experimental conditions with Ω_−_ = 2*π* · 8.33 MHz and *T*_DYSCO_ = 2.55 ms a theoretical maximum DR of 5 · 10^3^ is achievable.

## Discussion

The frequency and selectivity of a spin sensing protocol are governed by the filter function *F*(*ω*) characteristic of each pulse sequence. The *F*(*ω*) function is deduced from a sensitivity function *g*(*t*) that describes the instantaneous sensitivity of the spin during the evolution time^17^: $F(\omega )=\frac{{\omega }^{2}}{2}{| {\mathcal F} (g(t))|}^{2}$. The NV spin coherence signal *χ*(*t*) is influenced by the spin noise spectrum *S*(*ω*) in the environment and given by

4 $$\chi (t)={\int }_{0}^{\infty }\frac{{\rm{d}}\omega }{\pi }S(\omega )\frac{F(\omega t)}{{\omega }^{2}}$$

The filter functions *F*(*ω*) of the sensing protocols that accumulate a phase signal by means of free evolution intervals interleaved by *π*-pulses suffer from undesired harmonic responses as shown in Fig. 3(b)(left). The DYSCO protocol, however, provides harmonics free sensing, which can be explained in a spectroscopic representation using filter functions. As in DYSCO we modulate the dynamical sensitivity *β*(*t*) in a smooth way, the Fourier transform gives a single-valued frequency response (cf. Fig. 3(b) right). Either by sequentially scanning the desired frequency range or by non-uniform sampling we can perform weak precision magnetic sensing in the frequency domain^41, 42^.

The DYSCO method provides a piecewise generic spin control that can be incorporated into other pulse schemes. Some methods have been proposed to use cyclic phases^43^, correlation spectroscopy^44^ and non-uniform free-precession intervals^45, 46^ for suppressing harmonic responses. The DYSCO modulation units could be used to effectively tune the sensitivity function of these sequences and possibly extend the high frequency limits. Other schemes that employ active manipulation of nuclear spins^47^, spin-lock^48^ and Hartmann-Hahn polarization transfer^36, 49^ could avoid harmonic artefacts in sensing while being applicable in limited bandwidth naturally precluding multiplexing capabilities.

In summary, the DYSCO method provides a framework for preserving the sensitivity of a quantum system despite a noisy environment^24, 50^, while allowing for the modulation of its effective coupling to a desired external field in a controlled and robust fashion^20, 21^. Our method to boost the dynamic range and to allow sensing of weak signals without prior knowledge or control over the latter will benefit real-life applications of NV sensors^5–8^, while it can also be adopted for other quantum systems^26–28^. The DYSCO route to achieve *T*_1*ρ*_ limited sensing times and to detect sub-kHz coherent interactions without resorting to isotopically purified diamond provides accessible NV quantum technologies based on affordable materials^9^. While our results demonstrate decisive advantages of the DYSCO method in NV spin magnetometry, this method is seamlessly applicable for the measurement of other relevant physical quantities and even in other NV sensing modalities^51, 52^. The DYSCO method presented here provides a vital step towards realizing the full potential and uniqueness of the NV sensor as a tool for examining nanoscale phenomena and processes, addressing the challenges and technological demands of the future.

## Methods

### Sample and Setup

For the experiments we used a single NV centre in an electronic grade CVD grown diamond (Element Six). The spin manipulation is done in a home-built confocal microscope equipped with an arbitrary waveform generator. The schematics of our spin manipulation setup is shown in Supplementary Fig. S1.

In this study, we used a green laser at 532 nm to excite a single NV defect and collect the NIR fluorescence to determine the spin state population. A static magnetic field *B*_0_ is applied to lift the degeneracy of the spin sub-levels *m*_*s*_ = ±1. For our experiments we applied a field of *B*_0_ = 404 mT and used a MW frequency *ω*_−_ = 2*π* · 1737 MHz to drive spin transitions between the *m*_*s*_ = 0 and *m*_*s*_ = −1 ground state. A magnetic bias field is aligned parallel to the NV centre axis to perform dynamic nuclear polarization of the ^14^N spins. This results in an Optically Detected Magnetic Resonance (ODMR) line width of about 100 kHz without loosing the signal contrast. In our diamond sample, which contains natural abundance of 1.1% ^13^C nuclear spins, we selected an NV centre that did not show signatures of strongly coupled ^13^C nuclear spins.

The microwave and RF signals were generated using an arbitrary waveform generator (AWG; Tektronix 7122C) operating at 20 GS/s. The AWG conveniently synthesizes pulses with full frequency and phase control and reduces sources of pulse timing errors and phase delays, which might result from switches, phase shifters or combiners along the transmission line. Microwave synthesis via an AWG is, however, not indispensable; a conventional signal generator with additional IQ modulator would be able to perform the desired pulse control as well. The MW signal output of the AWG is connected to a 16 W microwave amplifier, and the power is fed into a copper wire with a diameter of around 20 *μ*m to drive the NV spin. This way, we achieve Rabi frequencies of Ω_−_ = 2*π* · 8.33 MHz. In addition, a small RF coil is placed in the vicinity of the sample that provides weak *B*_*RF*_ fields for magnetic field sensing experiments.

### Spectroscopy using DYSCO

**Figure 8:**
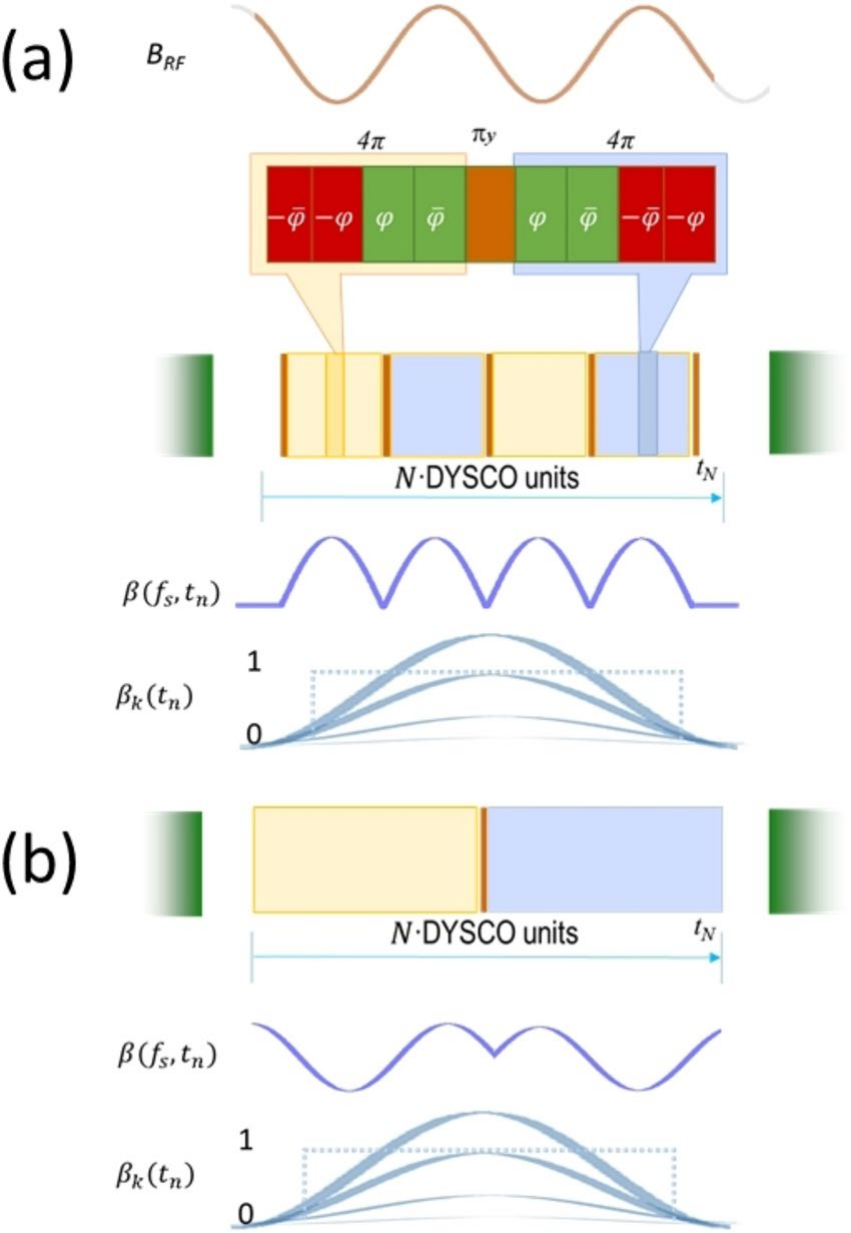
Schematic of two DYSCO modulation variants. (**a**) When the external RF field is phase synchronous with the applied MW pulses. (**b**) For sensing asynchronous RF noise signals.

For a measurement of the spectral components of a signal in the range of frequencies *f*_*s*_ ∈[*f*_min_, *f*_max_] with a resolution defined by the total sensing time *t*_*N*_, which is kept fixed. DYSCO spectroscopy is composed of a set of sequences one for every frequency *f*_*s*_ in the desired range. The DYSCO modulation pattern is unique for each frequency and symmetrical around the middle *π*_*y*_-pulse. The modulation pattern is naturally discretized by the 4 · *π*-pulse unit length, thus higher Rabi frequency can provide smoother modulation. The modulation is composed such that the dynamical sensitivity of the *n* ^th^ individual 4 · *π*-pulse unit is defined as *β*(*f*_*s*_, *t*_*n*_) = *β*_*k*_ sin (2*πf*_s_ *t*_*n*_) with *t*_*n*_ = (1 + 2*n*) · 2*π*/Ω_−_ and ∈ {0, 1, 2 …, *N*}. The amplitude factor *β*_*k*_ ∈ [0, 1] is constant for every *k*-th experimental run and can be stepwise increased with *k* ∈ {0, 1, …, *K*} which removes the typical |2*π*| ambiguity of interferometric sensing methods. Complete modulation periods are symmetrized by including *π*_*y*_-pulses, and if incommensurate portions are present they appear symmetrically. In the spectral domain, if the sin(*x*)/*x* wiggles are caused due to a rectangular window *β*_*k*_ = *k*/*K*, then by using a Gaussian envelop ${\beta }_{k}({t}_{n})=k/K\cdot \exp (-\frac{{({t}_{n}-{t}_{N}\mathrm{/2)}}^{2}\,}{\mathrm{2(}{t}_{N}{\mathrm{/2)}}^{2}})$ we can suppress these wiggles (cf. Fig. 8(a)).

Another variant of the sequence as displayed in Fig. 8(b) offers greater flexibility in a wide frequency span. The sequence is constructed with one *π*_*y*_ pulse in the middle of the evolution time and a number of *N* times the 4 · *π*-pulse modulation units (cf. Fig. 8(b)). The DYSCO noise spectrum is acquired as explained before in the frequency domain by measuring the signal *P*_0_ and probing a range of modulation frequencies and varying the maximum of the dynamical sensitivity *β*_*k*_. It should be noted that a single frequency scan with a fixed value of the sensitivity maximum *β*_*k*_ can also show the presence of spectral components, but without details on their magnitude.

### Comparison of DYSCO sensitivity to free-precision based schemes

To compare two methods under identical conditions, we set the spin evolution time *t*_*N*_ = 100 *μ*s and ramped the RF magnetic field. In case of Hahn-echo magnetometry this produced 42 population oscillations $({P}_{{m}_{s}=0}\leftrightarrow {P}_{{m}_{s}=-1})$. Under the same magnetic field change DYSCO magnetometry (at maximum sensitivity *β* = 1) produced 25 population oscillations. It can be seen the DYSCO sensitivity is reduced by ≈0.59 compared to free-precision. The analytical calculations detailing the results are given in the Supplementary Information S3ii).

## Electronic supplementary material

Supplementary Information
