# Supplementary material for: Dynamical sensitivity control of a single-spin quantum sensor
Source: Sci Rep. 2017 Jul 26;7:6586. doi: 10.1038/s41598-017-05387-w (PMC5529433; doi:10.1038/s41598-017-05387-w)
Supplement: Supplementary file 1 — Supplementary Information [file 41598_2017_5387_MOESM1_ESM.pdf]

Supplementary Information

Dynamical sensitivity control of a single-spin quantum sensor

Andrii Lazariev,[1,2] Silvia Arroyo-Camejo,[2] Ganesh Rahane,[1] Vinaya Kumar Kavatamane,[1] and Gopalakrishnan Balasubramanian[1,*]

[1]MPRG Nanoscale Spin Imaging, Max Planck Institute for Biophysical Chemistry, Am Fassberg 11, 37077 Göttingen, Germany

[2]Department of NanoBiophotonics, Max Planck Institute for Biophysical Chemistry, Am Fassberg 11, 37077 Göttingen, Germany

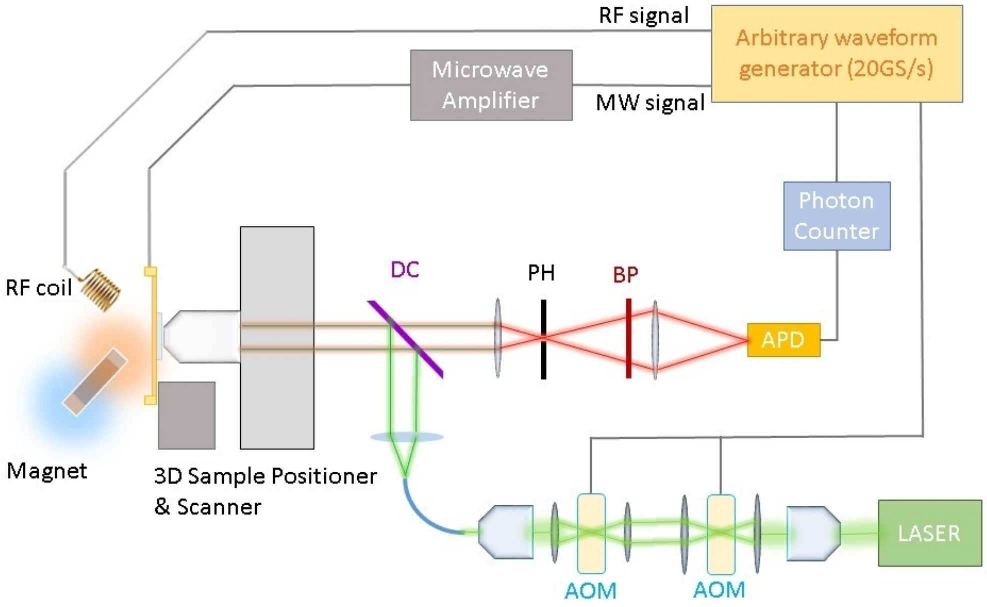

Figure. S1. Schematic of the setup used for single NV manipulation. A home-built confocal microscope equipped with control electronics (DC: Dichroic beam-splitter, PH: Pinhole, BP: Bandpass filter, AOM: Acousto-Optic Modulator; APD: Avalanche Photodiode). A computer interface and custom software are used to control the 3D scanner, arbitrary waveform generator, and the photon counter.

*Author to whom correspondence should be addressed: gbalasu@mpibpc.mpg.de

S1. NITROGEN-VACANCY DEFECTS IN DIAMOND

Nitrogen-vacancy (NV) centres are point defects in the diamond lattice that constitute of a substitutional nitrogen atom and a single vacancy forming a stable colour centre (cf. Fig. S2(a)). The NV centre we employ for this study is the negatively charged state (NV^-). The two unpaired electrons of the negatively charged NV centre form a spin-1 system featuring a triplet and a singlet level system. For our sensing application we employ the NV ground state triplet (cf. Fig. S2(b)) which is represented by the "bare system" Hamiltonian

$$H_{\mathrm{NV}} = DS_z^2 - \gamma_{\mathrm{NV}} B_0 S_z \ , \tag{S1}$$

where here and in the following we assume natural units ($\hbar = 1$), $D \approx 2\pi \cdot 2.87$ GHz is the zero field splitting, $\gamma_{\mathrm{NV}} \approx -2\pi \cdot 28$ GHz/T is the gyromagnetic ratio of the NV electron spin and $S = (S_x,\ S_y,\ S_z)$ is the spin-1 operator. The constant magnetic field B_0 is applied along the NV centre axis of symmetry to lift the degeneracy between the $m_s = \pm 1$ spin states. The NV centre ground state triplet level structure is illustrated in Fig. S2(b), showing the $m_s = 0$, the $m_s = +1$ and the $m_s = -1$ spin states, for which we use the alternative ket notation $|0\rangle$, $|+\rangle$, and $|-\rangle$, respectively, in the main article.

For controlled manipulation of the NV spin we apply a microwave field of amplitude $B_1^{(\pm)}$, frequency $\omega_{(\pm)}$ and phase $\varphi_{(\pm)}$ on resonance ($\delta_- = 0$) to one of the dipole allowed transitions of the NV centre: $|0\rangle$ and $|-\rangle$. The microwave field Hamiltonian applied to one of the two subspaces spanned by either $|0\rangle$ and $|+\rangle$ or $|0\rangle$ and $|-\rangle$ reads

$$H_{\mathrm{MW}}^{(\pm)}(t) = -\gamma_{\mathrm{NV}} B_1^{(\pm)} S_x^{(\pm)} \cos(\omega_\pm t + \varphi_\pm(t)) \ , \tag{S2}$$

which coherently drives the NV spin at a Rabi frequency of $\Omega_{(\pm)} = -\gamma_{\mathrm{NV}} B_1^{(\pm)}$ and were the $S_j^{(\pm)}$ with $j \in \{x,\ y,\ z\}$ are the spin-1/2 matrices defined on the two-dimensional subspaces spanned by $|0\rangle$ and $|+\rangle$ or $|0\rangle$ and $|-\rangle$.

We assume the external radio frequency field $B_{\mathrm{RF}}(t)$ to be aligned quasi-parallel to the NV centre axis, such that the respective Hamiltonian reads

$$H_{\mathrm{RF}}(t) = -\gamma_{\mathrm{NV}} B_{\mathrm{RF}}(t) S_z \ . \tag{S3}$$

The total system is now described by the Hamiltonian $H(t) = H_{\mathrm{NV}} + H_{\mathrm{MW}}^{(\pm)}(t) + H_{\mathrm{RF}}(t)$.

The ground states ^{3}A and excited states ^{3}E of the triplet system are shown in Fig S2(b). At room temperature the NV colour centre has a broad excitation and emission spectra. When excited

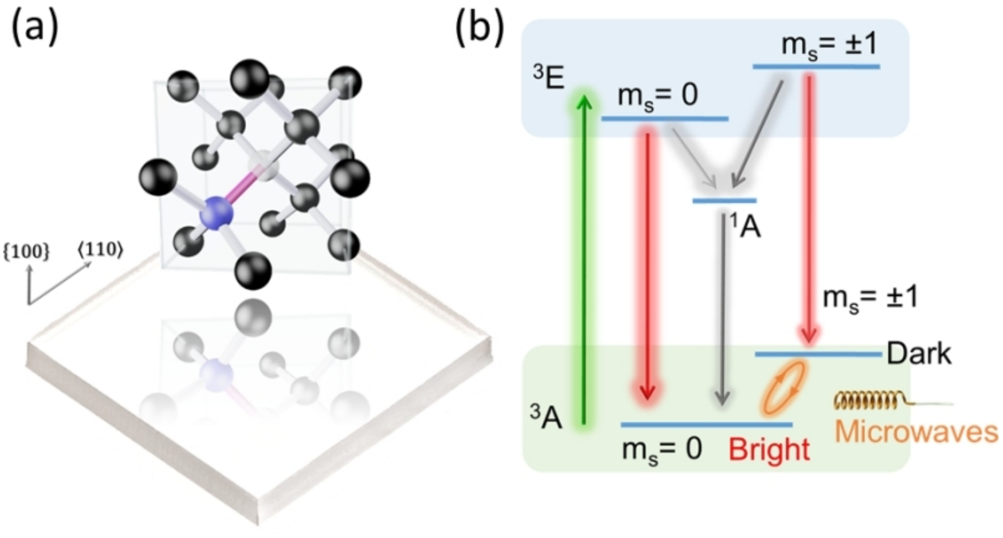

Figure. S2. (a) Illustration of an NV centre inside the unit cell of diamond. The NV centre employed in this work is embedded in an electronic grade bulk diamond slap (shown at the bottom). (b) Nitrogen-vacancy centre energy level scheme showing excitation and emission pathways. The green arrow indicates the 532 nm laser excitation (^{3}A to ^{3}E) while the red is fluorescence emission in the NIR (^{3}E to ^{3}A). Black arrows denote non-radiative routes through singlet states. Spin transitions in the ground states are coherently driven by microwave fields (orange) produced by an antenna.

by green light the NV centre decays back to the triplet ground state either by emission of a NIR photon or non-radiatively through singlet states. The inter-system crossing rate of the spin sub-levels $m_s = 0$ and $m_s = \pm 1$ are different, causing the $m_s = 0$ state to preferentially undergo spin-preserving, radiative decay, while the $m_s = \pm 1$ state undergoes non-radiative decay with a probability of 30%. For this reason the $m_s = \pm 1$ states appear "dark" if compared to the "bright" $m_s = 0$ state. Moreover, when the NV undergoes inter-system crossing the spin information is lost, resulting in optical polarization of the spin state into the $m_s = 0$ state.

S2. THEORETICAL ANALYSIS OF THE UNDERLYING MECHANISM OF DYSCO SPIN MANIPULATION

For a comprehensive analysis of the developed DYSCO sequence, here we follow two approaches. For small numbers of four π block repetitions (e.g., $N = 1$) the dynamics of the Bloch vector and its dependence on the external $B_{\mathrm{RF}}(t) = B_{\mathrm{RF}}^{(0)} \cos(\omega_{\mathrm{RF}} t + \varphi_{\mathrm{RF}})$ field as well as the phase angle φ

are directly computed from the system interaction Hamiltonian (in the rotating frame of the bare NV system)

$$H(t) = \hbar \begin{pmatrix} 0 & -\frac{\Omega_-}{2}\, e^{-i\varphi} \\ \frac{\Omega_-}{2}\, e^{-i\varphi} & -\delta_- + \gamma_{\mathrm{NV}} B_{\mathrm{RF}}(t) \end{pmatrix} . \tag{S4}$$

The spin evolution is obtained from the evolution operator

$$U(t,0) = \mathcal{T} e^{-i/\hbar \int_0^t H(t')\ \mathrm{d}t'} \tag{S5}$$

(where $\mathcal{T}$ denotes time ordering) by means of a custom written routine implemented in Wolfram Mathematica. In the following we present both numerical (see section S2 i) and analytical calculations (see section S3) explaining the underlying mechanism of the dynamical sensitivity control scheme.

i. Simulations of long DYSCO sequences using SpinDynamica

Here we show numerical simulations of the spin dynamics associated with the DYSCO sequence. As for large numbers N of the fundamental $4{\cdot}\pi$-pulse unit the evaluation becomes computationally challenging, here we resort to employ the SpinDynamica simulation package, which allows us to efficiently simulate typical, long experimental sequences with $N = 150$ up to $N = 200$.

For all simulations we consider the initial spin to be initialized to $|0\rangle$ and the spin driving microwave field is applied on resonance, i.e. $\delta_- = 0$. First we analyse the DYSCO sequence as presented in the main article:

$$\left[\pi_{\overline{x}-\varphi}\pi_{x-\varphi}\pi_{x+\varphi}\pi_{\overline{x}+\varphi}\right]^N - \pi_y - \left[\pi_{x+\varphi}\pi_{\overline{x}+\varphi}\pi_{\overline{x}-\varphi}\pi_{x-\varphi}\right]^N . \tag{S6}$$

Fig. S3(a) shows the Bloch vector evolution for each pulse of the DYSCO sequence in Eq. (S6), where the time evolution is colour coded for each pulse (blue indicates the start, red denotes the end of each pulse). The population $P_0(B_{\mathrm{RF}}, N)$ oscillates as a function of the external B_{RF} amplitude and the fast Fourier transform (FFT) of the function obtained for φ is denoted as $S(\zeta)$ and shown in Fig. S3(b) displaying the simulation results and c) showing experimental data. As described in the main article, we define $\arg\max_{\zeta}|_{\varphi}$ of this quantity as dynamical sensitivity $\beta(\varphi, B_{\mathrm{RF}}, N)$. Fig. S3(b) and (c) demonstrate that simulations and experiments correspond very well.

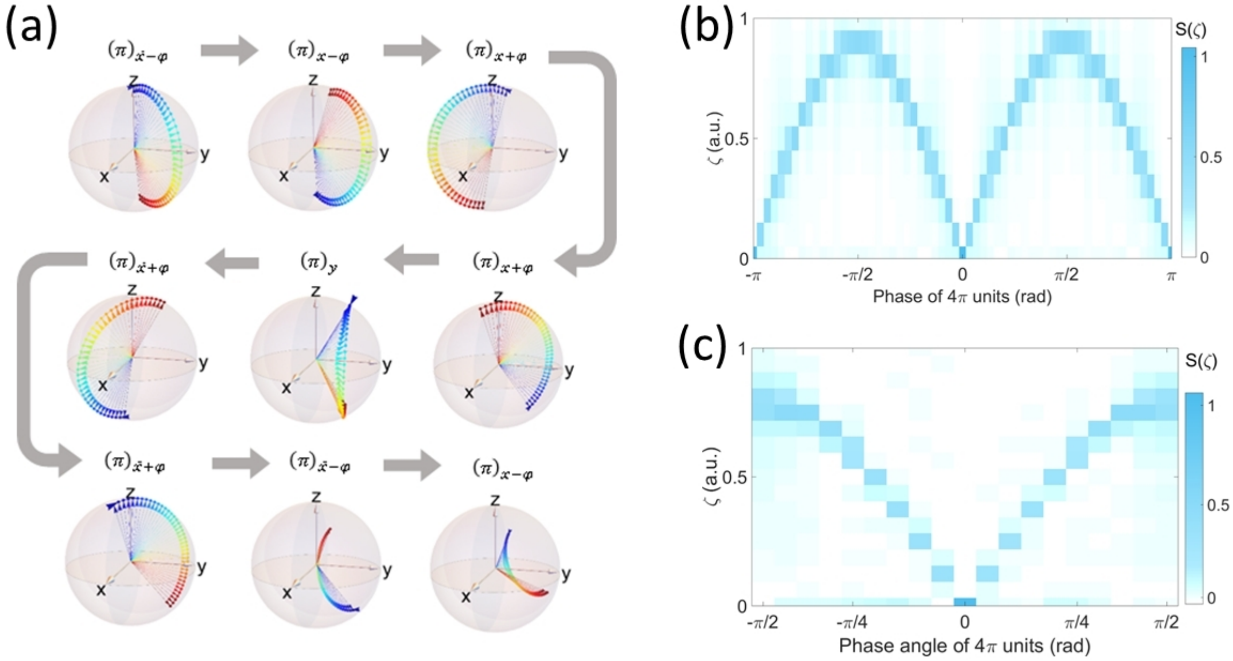

Figure. S3. Bloch sphere dynamics, simulation and experimental results of the DYSCO pulse sequence from Eq. (S6) simulated using SpinDynamica. (a) The pulse-by-pulse Bloch vector dynamics for one full sequence. (b) Simulations showing the FFT of the P_0 dependence on B_{RF} and φ for $N = 160$ units. (c) Experimental results of $S(\zeta)$ and the dynamical sensitivity β variation with pulse phase angle φ for a set of $N = 200$ units. The initial state starts from the bright state $m_s = 0$.

ii. Simulations for DYSCO sequence with first and last $(\pi/2)$ pulses

To demonstrate that the DYSCO scheme relies only on the population change without being specific to any particular state, we present a slightly modified version of the DYSCO sequence from Eq. (S6). In this variant we surround the DYSCO pulse sequence with a pair of $\pi/2$ pulses represented in the following way:

$$\left(\frac{\pi}{2}\right)_x - [\pi_{\overline{x}-\varphi}\pi_{x-\varphi}\pi_{x+\varphi}\pi_{\overline{x}+\varphi}]^N - \pi_y - [\pi_{x+\varphi}\pi_{\overline{x}+\varphi}\pi_{\overline{x}-\varphi}\pi_{x-\varphi}]^N - \left(\frac{\pi}{2}\right)_x \tag{S7}$$

Similar to the previous section, Fig. S4(a) shows the Bloch vector evolution for each pulse of the DYSCO sequence in Eq. (S6) in colour code. We observe that the increase in the B_{RF} field gives raise to oscillation in the population $P_0(B_{\mathrm{RF}}, N)$ and the corresponding FFT is denoted by $S(\zeta)$ and is shown in Fig. S4(b) and (c), displaying the simulation and experimental results, respectively.

Fig. S4(b) and (c) show that simulations and experiments agree well. The dependence is similar to that obtained when initial state starting from the pure state $m_s = 0$.

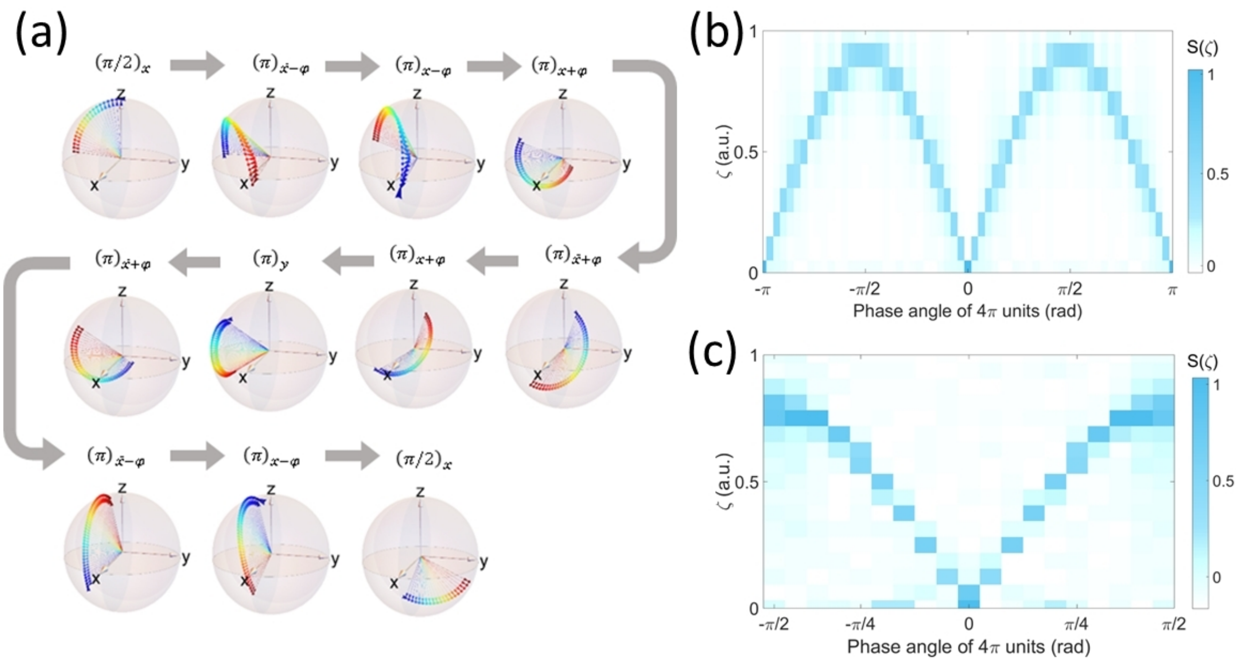

Figure. S4. Bloch sphere dynamics, simulation and experimental results of the DYSCO pulse sequence from Eq. (S7) simulated using SpinDynamica. (a) The pulse-by-pulse Bloch vector dynamics for one full sequence. (b) Simulations showing $S(\zeta)$ the FFT of the P_0 dependence on B_{RF} and φ for $N = 160$ units. (c) Experimental results of $S(\zeta)$ and the dynamical sensitivity β variation with pulse phase angle φ for a set of $N = 200$ units.

S3. ANALYTICAL APPROXIMATION OF THE DYSCO SPIN DYNAMICS FOR SMALL MAGNETIC FIELD

In addition to the numerical simulation presented above, here we perform analytical approximations valid for small RF magnetic field amplitudes $B_{\mathrm{RF}}^{(0)}$ and vanishing microwave detuning $\delta_- = 0$ from resonance. This way we derive an explicit analytical expressions for the dependence of the occurring population shift as a function of the RF magnetic field amplitude $B_{\mathrm{RF}}^{(0)}$ with respect to the driving Rabi frequency field B_1.

Fig. S5 illustrates the Bloch sphere including the applied angular momentum vectors associated

with the external RF magnetic field B_{RF} as well as the driving microwave field. In the following we will drop the index "1" of the microwave driving field B_1 and replace it by B_ξ , when referring to a specific microwave pulse of phase ξ.

The joint action of the B_{RF} and B_ξ fields on the Bloch vector can be described by the rotation matrix

$$M_{(\varphi,\theta,\gamma)} = \begin{pmatrix} \mathrm{v}\gamma \mathrm{s}^2\theta \mathrm{c}^2\varphi + \mathrm{c}\gamma & \mathrm{v}\gamma \mathrm{s}^2\theta \mathrm{c}\varphi \mathrm{s}\varphi - \mathrm{s}\gamma \mathrm{c}\theta & \mathrm{v}\gamma \mathrm{s}\theta \mathrm{c}\theta \mathrm{c}\varphi + \mathrm{s}\gamma \mathrm{s}\theta \mathrm{s}\varphi \\ \mathrm{v}\gamma \mathrm{s}^2\theta \mathrm{c}\varphi \mathrm{s}\varphi + \mathrm{s}\gamma \mathrm{c}\theta & \mathrm{v}\gamma \mathrm{s}^2\theta \mathrm{s}^2\varphi + \mathrm{c}\gamma & \mathrm{v}\gamma \mathrm{s}\theta \mathrm{c}\theta \mathrm{s}\varphi - \mathrm{s}\gamma \mathrm{s}\theta \mathrm{c}\varphi \\ \mathrm{v}\gamma \mathrm{s}\theta \mathrm{c}\theta \mathrm{c}\varphi - \mathrm{s}\gamma \mathrm{s}\theta \mathrm{s}\varphi & \mathrm{v}\gamma \mathrm{s}\theta \mathrm{c}\theta \mathrm{s}\varphi + \mathrm{s}\gamma \mathrm{s}\theta \mathrm{c}\varphi & \mathrm{v}\gamma \mathrm{c}^2\theta + \mathrm{c}\gamma \end{pmatrix} \tag{S8}$$

performing a rotation of the Bloch vector by an angle γ around the axis specified by spherical coordinates (φ, θ), where we use the abbreviations $\mathrm{v}\gamma = (1 - \cos\gamma)$, $\mathrm{c}\theta = \cos\theta$ and $\mathrm{s}\theta = \sin\theta$. After application of the DYSCO pulse sequence to the initial Bloch vector $\vec{b}_{\mathrm{ini}}$ the final Bloch vector is given as

$$\vec{b}_{\mathrm{final}} = M_{\mathrm{DYSCO}}\, \vec{b}_{\mathrm{ini}} \tag{S9}$$

where the transformation matrix M_{DYSCO} is given as the time-ordered product of the rotation matrices of the $(2N+1)$ individual pulses

$$M_{\mathrm{DYSCO}} = \prod_{i=1}^{2N+1} M_{(\varphi_i,\theta_i,\gamma_i)} \; . \tag{S10}$$

The population of the $|0\rangle$ and the $|-\rangle$ states are then readily obtained from the z-axis component b_z of the final Bloch vector as $P_{(0,-)} = 1/2(1 \pm b_z)$.

For the DYSCO sequence starting from the Bloch sphere pole from Eq. (S6) we obtain the population of the $|0\rangle$ state as a Taylor expansion given by

$$P_0 = 64\frac{B_{\mathrm{RF}}^2}{B_1^2}\sin^2\varphi + \mathcal{O}(B_{\mathrm{RF}})^4 \; . \tag{S11}$$

For the DYSCO sequence starting from the equatorial plane by using a $\pi/2$ pulse described in Eq. (S7) we analogously obtain the Taylor expansion as

$$P_0 = \frac{1}{2} - 8\frac{B_{\mathrm{RF}}}{B_1}\sin\varphi + \mathcal{O}(B_{\mathrm{RF}})^3 \; . \tag{S12}$$

The dynamical sensitivity $\beta(\varphi)$ of a sequence can be deduced from these expressions for P_0. We observe that the characteristic dependence of the dynamical sensitivity obtained for the DYSCO sequence initialized along the pole as in Eq. (S6) and for the case initialized along the superposition plane Eq. (S7) are similar.

i. Visualization of the DYSCO spin-dynamics on a Bloch sphere for small magnetic field

We will present an intuitive illustration and analyse the dynamics of the spin vector upon application of the DYSCO sequence that is described in Eq. (S7). After the spin had be initialized to $|0\rangle$ (i.e., the north pole of the Bloch sphere) the initial $\left(\frac{\pi}{2}\right)_x$ pulse drives the Bloch vector to point into the $-y$-axis direction. Now the application of the first four π-pulse block $[\pi_{\overline{x}-\varphi}\pi_{x-\varphi}\pi_{x+\varphi}\pi_{\overline{x}+\varphi}]$ leads to a zig-zag trajectory on the Bloch sphere. Note that while $B_{\overline{x}-\varphi}$ and $B_{x-\varphi}$ are pointing to opposite directions, the resulting rotation axes $a_{\overline{x}-\varphi}$ and $a_{x-\varphi}$ are not collinear, owing to the influence of the $B_{\rm RF}$ magnetic field. After every second π pulse the spin comes back (close) to the yz-plane (i.e., the x-component of the Bloch vector is very small).

The position of the spin vector after the four π-pulse block is then obtained as

$$\begin{pmatrix} x \\ y \\ z \end{pmatrix} = \mathbf{M}_{(\overline{x}+\varphi,\theta,\pi)}\mathbf{M}_{(x+\varphi,\theta,\pi)}\mathbf{M}_{(x-\varphi,\theta,\pi)}\mathbf{M}_{(\overline{x}-\varphi,\theta,\pi)} \begin{pmatrix} 0 \\ -1 \\ 0 \end{pmatrix} \tag{S13}$$

where the rotation axis of the Bloch vector is specified by the pulse phase φ and the angle $\theta = \frac{\pi}{2} - \arcsin\left(\frac{B_{\rm RF}}{B_1}\right)$ (i.e., the ratio between RF field and driving field B_1) as illustrated in Fig. S5. If we approximate the resulting expression from Eq. (S13) for small RF magnetic fields (i.e., $B_{\rm RF} \ll B_1$), the small angle approximation delivers $\theta \approx \frac{\pi}{2} - \frac{B_{\rm RF}}{B_1}$. In this case the z-component of the resulting vector will be similar to the difference in polar angle

$$\Delta\theta \approx -\frac{1}{4}\left(2\sin(4\theta) + 3\sin(8\theta)\right)\sin(\varphi) - 2\sin^3(2\theta)\cos(2\theta)\sin(3\varphi) \tag{S14}$$

which is acquired over the run of the four π-pulse sequence. From a Taylor expansion of this expression up to second order in $B_{\rm RF}$ we finally obtain the explicit expression for the polar angle shift as

$$\Delta\theta \approx 8\frac{B_{\rm RF}}{B_1}\sin\varphi + \mathcal{O}(B_{\rm RF})^3 \ . \tag{S15}$$

The derived sine dependence of the Bloch vector polar angle difference $\Delta\theta$ (and thus also of the population shift $\Delta P_0 = 1/2(1+\cos\theta) \approx 1/2(1+\Delta\theta))$ from the phase angle φ of the pulse sequence is in close correspondence to the numerical simulations and experimental results presented in the previous section. The latter show that the sine dependence of the acquired phase (per four π-pulse block) is valid not only for the DYSCO variant from Eq. (S7) starting from the equatorial plane, but also for the original DYSCO sequence from Eq. (S6) starting from the pole of the Bloch sphere.

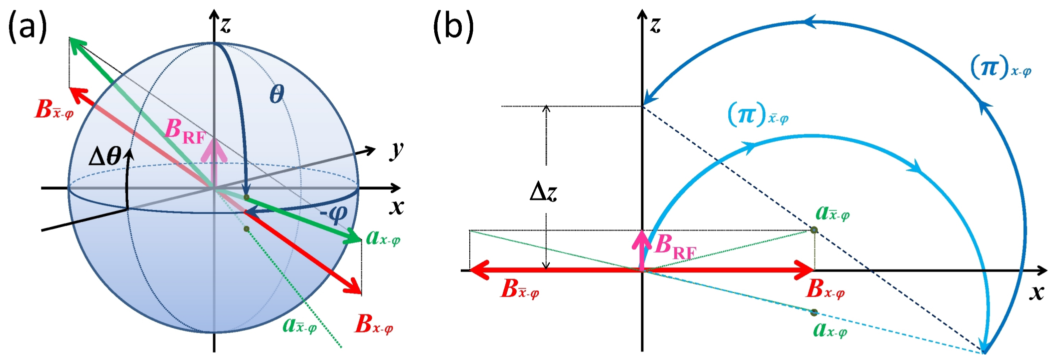

Figure. S5. (a) Representation of the Bloch sphere and the applied angular momentum vectors associated with the external RF magnetic field B_{RF} and the driving microwave fields B_ξ. Note $B_{\overline{x}-\varphi}$ and $B_{x-\varphi}$ denote the directions of microwave driving field B_1 used for the first two π-pulses of the four π-pulse block. In the approximation $B_{\mathrm{RF}} \ll B_1$ the resulting shift of the Bloch vector polar angle $\Delta\theta$ corresponds to the change Δz of the Bloch vector z-component.
(b) Projection onto the xz-plane of the Bloch vector during the first two π-pulses of the four π-pulse block: the first rotation along the a_φ axis and the following along the $a_{-\phi}$ axis bring the Bloch vector out of equatorial plane of the Bloch sphere, resulting in a shift of the Bloch vector z-component.

These results also agree with the analytical expression in Eq. (S12) obtained for the complete 9π DYSCO pulse sequence.

Here, we analytically derived that the DYSCO sequence produces a magnetic field dependent population shift (associated to a change $\Delta\theta$) of the Bloch vector polar angle, instead of a magnetic field dependent phase shift (i.e. a change $\Delta\phi$ of the Bloch vector azimuthal angle) as in the case of multi-pulse sequences. We show that the spin dynamics and the mechanism associated with the DYSCO sensing scheme are distinct and they complement free-precession based sensing schemes that primarily accumulate phase on the equatorial plane of the Bloch sphere (e.g. Ramsey, Hahn-echo or CMPG sequences).

ii. Sensitivity comparison of DYSCO and free precession sensing schemes

In order to compare the sensitivity of the DYSCO scheme with the sensitivity of free precession sensing schemes, we will calculate the deviation angle of the Bloch vector from its initial direction for the both cases. In the DYSCO sensing scheme, one block of the pulse sequence induces the vector angle change as calculated in Eq.(S15). Assuming the maximal sensitivity condition ($\sin\varphi = 1$),

one obtains

$$\Delta\theta = 8\frac{B_{\mathrm{RF}}}{B_1} \ , \tag{S16}$$

where B_{RF} is the sensed magnetic field and B_1 is the driving field. The evolution time for the application of one DYSCO block, i.e. four π-pulses, can be expressed through the driving field as four times

$$t_\pi = \frac{T_R}{2} = \frac{\pi}{\Omega_R} = \frac{\pi}{\gamma B_1} \ , \tag{S17}$$

where T_R denotes the period of the Rabi oscillation and Ω_R is the angular Rabi frequency.

During the same period of time, the free precessing spin will acquire a phase of

$$\Delta\phi = \omega \cdot 4t_\pi = 4\pi\frac{B_{\mathrm{RF}}}{B_1} \ , \tag{S18}$$

where $\omega = \gamma B_{\mathrm{RF}}$.

The ratio of the angles from Eq.(S16) and Eq.(S18) is the ratio of sensitivities of the respective sensing schemes

$$\frac{\Delta\theta}{\Delta\phi} = \frac{2}{\pi} \approx 0.64. \tag{S19}$$

Comparing the sensitivities obtained experimentally (25 population oscillations in DYSCO vs. 42 population oscillations in Hahn-Echo for the same B_{RF} amplitude ramp, refer to Sec. "Comparison of DYSCO sensitivity to free-precision based schemes") we obtain a ratio of $25/42 \approx 0.6$. This value is in a good correspondence with the theoretical calculation.
